# The National Football League and traumatic brain injury: blood-based evaluation at the game

**DOI:** 10.2217/cnc-2023-0011

**Published:** 2023-09-13

**Authors:** Steven H Rauchman, Dimitris G Placantonakis, Allison B Reiss

**Affiliations:** 1 The Fresno Institute of Neuroscience, Fresno, CA 93730, USA; 2 Department of Neurosurgery, NYU Grossman School of Medicine, New York, NY 10016, USA; 3 Department of Medicine & Biomedical Research Institute, NYU Grossman Long Island School of Medicine, Mineola, NY 11501, USA

**Keywords:** biomarker, brain injury, diagnostic and management tests, return-to-play, sports-related concussion

## Abstract

#brain #injury in the #football #player - we need better #diagnosis and #prevention. #view our #latest #publication in the #journal Concussion @futuresciencegp on @thegame #Blood test #biomarker #innovation #safety @NFL

Concussions continue to be a major problem facing contact sports organizations, particularly the National Football League (NFL) [1,2]. The NFL, NFL Players Association, and medical experts from multiple clinical disciplines have authored detailed protocols for reviewing and managing head injuries [1,3]. Head injury to NFL athletes has been the topic of extensive reviews in the academic literature [4]. Risk for sustaining a concussion while playing football has tended to decrease over time, but is still substantial. Concussion risk during the 2015 NFL season was about 8% while during the 2019 NFL season it had fallen to about 7.1% [5].

There are long-term consequences to cognitive performance in individuals experiencing sports-related concussions. Retired athletes who experienced sports-related concussions had a decrease in cognitive performance. The onset of chronic traumatic encephalopathy (CTE) in NFL players has previously been recognized [6]. Moreover, a highly significant portion of post-mortem exams of ex-NFL players demonstrate evidence of CTE.

The NFL has invested resources in an attempt to prevent head injuries. The area of helmet design has been extensively investigated [7]. Rules have been strengthened to protect players from concussions, notably spurred on by the recent injury to Tua Tagovailoa in 2022.

A detailed protocol related to concussions includes a club-designated neuropsychologist consultant to do periodic preseason baseline neurocognitive testing and retests after head trauma [1]. Game day observations include spotters at locations around the field for injuries that might be missed by the sidelines. There is a rapid replay video system. If a player is identified with an injury, there is a standardized protocol administered immediately in a sideline blue tent. For suspected concussions, the player is taken to the locker room. Athletes are then followed in a step-by-step process following game day for an extended period in a return-to-play protocol. However, there are currently no routine blood biomarker screening tests in this lengthy protocol. Concussion remains a clinical diagnosis with no definitive test to distinguish concussed from non-concussed individuals [8]. We highlight here that there is now a simple, validated diagnostic test to determine whether a concussion or traumatic brain injury (TBI) has occurred.

Neuroimaging often takes place in the Emergency Department, but routine scans in patients suffering concussions or TBI are usually negative for intracranial bleeding. Cellular damage from acute head injury does lead to the release of GFAP and UCH-L1, two proteins that can serve as biomarkers of brain trauma [9]. The prognostic value of measuring plasma levels of GFAP and UCH-L1 in same day evaluation of head injury has been demonstrated with good-to-excellent accuracy for predicting death and unfavorable outcomes, but not for predicting incomplete recovery at 6 months [10]. Bazarian *et al.* [11] evaluated the accuracy of a blood test combining measurements of both GFAP and UCH-L1 for predicting acute traumatic intracranial injury (TII) on head CT after mild TBI. All evaluated subjects presenting to emergency departments with non-penetrating head trauma had routine head CTs and blood sampling within 12 hours of injury. Plasma GFAP and UCH-L1 were measured using i-STAT Alinity and TBI cartridge (Abbott labs) and compared to acute TII on head CT. The rapid blood test had a sensitivity of 0.958, specificity 0.404, negative predictive value of 0.993 and positive predictive value of 0.098 for acute TII. The conclusion was that a rapid i-STAT based test had high sensitivity for prediction of acute TII.

Another important study examined the impact of these markers within the first 12 months in military-related TBI [12]. GFAP predicted the deterioration of post-concussion post-traumatic stress disorder, depression, anxiety, somatic, neurologic and cognitive symptoms. UCH-L1 predicted the worsening of anxiety, somatic and neurologic symptoms. The value of such bio-measurements during the first week after TBI has been clarified [13]. Results support the utility of GFAP and UCH-L1 in acute phase diagnostics of TBI.

The FDA has now approved Abbott's Alinity i TBI lab test, the first commercially available point-of-care mild TBI test [14]. It offers a reliable result in 18 minutes to help clinicians quickly assess individuals with possible concussion. This test was approved to screen patients with head trauma to avoid unnecessary CT scans, which is a valuable goal in saving medical expense and avoiding unnecessary radiation exposure. Further, this lab test may outperform CT scans in mild TBI. In our opinion, there is value in applying this blood test for NFL players who routinely experience head trauma. This is a cost-effective solution that the NFL could adopt in future.

This paper examines a subject involving immense public interest, economic impact, and personal health with the potential to modulate long-term cognitive outcomes. The subject of head injuries in the NFL is not new. Football is a tremendously popular contact sport where concussions are inevitable, despite helmets and other protections. Rule modifications such as targeting prohibitions aim to mitigate against the most serious injuries. Improving headgear helps, but there are limits to what modifications are technically possible while still maintaining player comfort and performance [15]. The NFL has organized medical experts from a variety of specialties to design safety protocols to screen players with potential concussion. New technology and additional human resources have been made available at the sidelines. Player and coaching education has been instituted.

Unfortunately, players continue to sustain significant head trauma [16]. Long-term consequences of those repeated blows are now well-known in the medical community and to the general public. CTE is a haunting reminder of what a player may experience down the road. The stories of relatively young retired NFL veterans in cognitive and emotional decline are all too common. There is no objective test to determine if a person has had a concussion or mild TBI after head injury. Cognitive assessments can be made and questions asked, but these are not precise tools [17].

GFAP and UCH-L1 are released from the brain during head injury. These two brain injury markers have been scientifically validated and can be easily measured in peripheral blood [18,19]. Their quantitation is the basis for an FDA-approved lab test that is currently commercially available. This test can be done at relatively low cost with rapid turnover of results (18 minutes).

We suggest that, as the 2023–2024 NFL season begins, pilot studies be initiated to determine whether this test should be added to the NFL head injury protocol. The blood could be drawn in the concussion tent or locker room. This is simple and practical. The lab assessment could be done immediately; Abbott and the NFL could certainly work out any necessary accommodations. A committee of medical experts should be assembled to incorporate blood test results into established protocols. It is not the intent of this article to dictate exactly how the results should be used. One point is, however, difficult to debate. Any player testing positive should be immediately excluded from return to play. This one added stipulation could preserve the future cognitive health of NFL players. If a player demonstrates documented release of brain-derived products in his blood, then further exposure to trauma and repeated head injury must be avoided.

One could argue that the blood test might be done too early after head trauma and miss the positivity window. This outcome is recognized as a limitation. A negative test is not a guarantee of well-being. A negative blood result should be employed by trained medical providers to make a holistic clinical decision in context. A player may still be excluded from play if other neurologic signs of significant head trauma are present. The blood test may need to be repeated.

The test results might have other far-ranging implications. Even if the blood sample is not immediately drawn during the game, it could be obtained at halftime or at the conclusion of play. The test may have predictive value moving forward longitudinally. The test might have implications on return to play the following week. Serial measurements over time might prove value for longer-term decision-making. Systematic follow-up of athletes who test positive or negative (including the actual quantitative result) might provide long-term medical and health-risk information. If a large database is established, then future NFL players may be able to make more data-driven choices about the trajectory of their careers [20].

NFL players are an easily studied population of individuals who experience TBI and have access to ongoing and high-level medical care. The results of TBI among NFL players might be relevant for those acquired during other sports, such as soccer, and to military TBI as well. Thus, the Department of Defense could then arrive at more informed decisions on how to treat military personnel after head trauma. The societal benefits could be important. Most individuals experience TBI from falls or motor vehicle accidents. Those patients are often lost to follow-up and may benefit from biomarker blood tests.

In conclusion, we urge the NFL to consider piloting the integration of the Abbott biomarker blood test into existing protocols. As other blood tests receive approval, they can be considered for this type of application. This may open a new era of more carefully defined TBI research. At the very least, current NFL players may realize immediate benefits.
